# Editorial: Weak interactions in molecular machinery volume II

**DOI:** 10.3389/fmolb.2023.1284353

**Published:** 2023-09-15

**Authors:** Laura Corrales-Guerrero, Filippo Prischi, Irene Díaz-Moreno

**Affiliations:** ^1^ cicCartuja, Institute for Chemical Research (IIQ), University of Seville—CSIC, Seville, Spain; ^2^ Randall Centre for Cell and Molecular Biophysics, King’s College London, London, United Kingdom

**Keywords:** weak interaction, structural biology, biochemistry, biophysics, fuzzy ensemble

Weak molecular interactions within cells constitute a captivating realm of research that has gained unprecedented insight and momentum in recent years. These subtle forces play critical roles in protein folding, enzymatic catalysis, molecular recognition, DNA replication and repair, membrane dynamics, cellular assembly, and drug discovery. By facilitating rapid and reversible responses, these interactions enable cells to maintain structure, execute intricate signaling pathways, and carry out diverse processes, with implications for both understanding fundamental biology and developing innovative therapeutic strategies. Intriguingly, weak interactions operate in concert, collectively influencing the behavior of cells. Weak interactions can be highly specific, determining the selectivity of protein-protein interactions, enzyme-substrate recognition, and ligand binding. Moreover, they are dynamic, allowing for rapid responses to changing conditions within the cellular environment. As our understanding of these interactions deepens, innovative techniques have emerged, revolutionizing the way we study and manipulate these fundamental processes. In this Research Topic, we collected a series of articles that explore transient interactions in the cell, illustrating some examples where biophysics, structural or molecular dynamics approaches were used to unravel the molecular mechanisms beyond such processes.

Cells are crowded environments where a myriad of biomolecules, including proteins, nucleic acids, and other cellular components, coexist within a limited volume. This molecular crowding has significant implications for cellular processes, affecting molecular interactions, diffusion rates, and overall cellular homeostasis. Indeed, the concentration of different biomolecules is not homogeneous across cells, yet it is a highly controlled phenomena regulating cellular processes in time and space. Using molecular dynamics (MD) simulations, Jenkins et al. delve into the effects of cellular crowding on the protein-protein energy funnel dimensions. They uncover a fascinating phenomenon wherein the funnel size shrinks as protein concentrations rise, resulting in potential modifications in protein-protein interactions and overall functionality. This narrowing of the energy funnel in crowded cellular conditions could influence the folding landscape and binding kinetics of proteins, subsequently impacting critical cellular processes, contributing to a deeper understanding of how cellular crowding alters the biophysical behavior of proteins.

Nearly all biomolecules in nature are able to perform a function by interacting with other biomolecules, sometimes as part of intricate networks. Structural biology hybrid methods, which combines and integrates experimental structural biology approaches with biophysical, biochemical and computational methods, have allowed to dissect molecular interaction at atomic level, providing vital new information about regulation of biomolecular complexes. The work by Thapa et al. describes the interaction between Protein phosphatase 2A (PP2A) and its inhibitor ARPP-19, which recently have been shown to play a key role in several human cancer types. Using nuclear magnetic resonance (NMR) spectroscopy and small-angle X-ray scattering (SAXS), they structurally characterized ARPP-19 and its variant ARPP-16, revealing that they are intrinsically disordered but contain some secondary structure elements. Microscale thermophoresis (MST) and NMR showed that ARPP binds PP2A A-subunit through linear motifs with modest affinity, while B56-subunit interaction was weak and transient. These findings establish a new basis for future studies and the generation of therapeutic interventions targeting ARPP-PP2A interactions. Ming et al. investigated how iron and copper ions affect ferritin, a universal iron storage and transporter protein, in the blood clam *Tegillarca granosa*. X-ray data on crystallized protein revealed changes in ferritin behavior due to these interactions. Iron binding influences ferritin conformation, potentially affecting iron storage and release. Conversely, copper could contact the ferroxidase site over Fe^2+^ ions, likely inhibiting the ferroxidase activity of the protein, with regulatory implications. Understanding these effects enhances our knowledge of ferritin role in metal ion regulation within the clam, offering implications for both iron storage and copper balance mechanisms. Similarly, Gilep et al. explores the diversity of 3Fe-4S ferredoxins in *M. tuberculosis*, providing detailed structural insights through X-ray crystallography. Of note, they crystallized for the first time the complex between a 3Fe-4S ferredoxin and P450, revealing crucial insights into their interaction and functional interplay. The high-affinity complex exhibits a certain degree of flexibility, as revealed by SPR and SAXS analyses. The findings of the study illuminate the versatility of these ferredoxins in mediating electron transfer processes and underline their significance in the context of *Mycobacterium tuberculosis* biology. This comprehensive exploration enhances our understanding of the intricate molecular mechanisms underlying redox reactions involving ferredoxins and P450, potentially paving the way for targeted interventions against tuberculosis. In another study investigating the complex nature of protein-cofactor interactions, Rivero et al. examine the formation of a complex between riboflavin kinase and pyridoxine 5′-phosphate oxidase, shedding light on their association in delivering the flavin cofactor. Their calorimetric and kinetics studies elegantly show the transient nature of the interactions, suggesting a dynamic and temporary association for efficient cofactor transfer. The advances of the study, achieved through a set of biophysics analyses, biochemical experiments, and MD simulations, offer a deeper understanding of the molecular mechanisms involved in FMN cofactor delivery, potentially contributing to the development of strategies targeting these interactions for therapeutic applications.

Particular efforts have been dedicated in recent years to further develop structural biology approaches to exploit biological information for the design, identification and development of chemical probes, ligands and inhibitors. Hough et al. discuss the benefits of employing X-ray diffraction techniques to determine the structures of protein-ligand complexes at room temperature over classical X-ray crystallography. This approach offers a more biologically relevant perspective by capturing the dynamic nature of these interactions under conditions closer to physiological settings compared to traditional cryogenic methods. By facilitating the study of molecular recognition and drug design in a native-like environment, room temperature X-ray diffraction holds promise for advancing our comprehension of complex biological processes and enhancing drug development approaches.

In conclusion, the study of weak molecular interactions within cells has undergone a transformative journey in recent years, driven by cutting-edge techniques that provide unprecedented insights into these fundamental processes. To that end, this Research Topic beautifully recapitulates the compendium of techniques available up to date for investigating such a dynamic process. It is clear that the toolbox of techniques will continue to expand, likely unveiling even deeper the role of weak molecular interactions in cellular life, with implications spanning from basic research to the development of innovative therapies.

